# Health-related quality of life of child and adolescent retinoblastoma survivors in the Netherlands

**DOI:** 10.1186/1477-7525-5-65

**Published:** 2007-12-03

**Authors:** Jennifer van Dijk, Jaap Huisman, Annette C Moll, Antoinette YN Schouten-van Meeteren, Pieter D Bezemer, Peter J Ringens, Peggy T Cohen-Kettenis, Saskia M Imhof

**Affiliations:** 1 Department of Medical Psychology, VU University Medical Center Amsterdam, the Netherlands; 2 Department of Ophthalmology, VU University Medical Center Amsterdam, the Netherlands; 3 Department of Pediatric Oncology, VU University Medical Center, Amsterdam, the Netherlands; 4 Emma Children's Hospital, Academic Medical Center, Amsterdam, the Netherlands; 5 Department of Clinical Epidemiology and Biostatistics, VU University Medical Center Amsterdam, the Netherlands

## Abstract

**Background:**

To assess health-related quality of life (HRQoL) in children (8–11 years) and adolescents (12–18 years) who survived retinoblastoma (RB), by means of the KIDSCREEN self-report questionnaire and the proxy-report version.

**Methods:**

This population-based cross-sectional study (participation rate 70%) involved 65 RB survivors (8–18 years) and their parents. Child/adolescents' and parents' perception of their youth's HRQoL was assessed using the KIDSCREEN, and the results were compared with Dutch reference data. Relations with gender, age, marital status of the parents, and visual acuity were analyzed.

**Results:**

RB survivors reported better HRQoL than did the Dutch reference group on the dimensions "moods and emotions" and "autonomy". Increased ratings of HRQoL in RB survivors were mainly seen in perceptions of the younger children and adolescent girls. RB survivors with normal visual acuity scored higher on "physical well-being" than visually impaired survivors. Age was negatively associated with the dimensions "psychological well-being", "self-perception" (according to the child and parent reports) and "parent relations and home life" (according to the child). "Self-perception" was also negatively associated with visual acuity (according to the child). Only parents of young boys surviving RB reported lower on "autonomy" than the reference group, and parents of low visual acuity and blind RB survivors reported higher on "autonomy" than parents of visually unimpaired survivors. Survivors' perceptions and parents' perceptions correlated poorly on all HRQoL dimensions.

**Conclusion:**

RB survivors reported a very good HRQoL compared with the Dutch reference group. The perceptions related to HRQoL differ substantially between parents and their children, i.e. parents judge the HRQoL of their child to be relatively poorer. Although the results are reassuring, additional factors of HRQoL that may have more specific relevance, such as psychological factors or coping skills, should be explored.

## Background

Retinoblastoma (RB) is a malignant tumor affecting the retina and is the most common intraocular malignancy in children. In the Netherlands, the incidence is 1:17,000 newborns (approximately 10–15 new patients every year)[1]. The survival of children with RB has significantly improved to a 5-year disease-free rate of more than 90% in the western world [2]. RB can affect one or two eyes, with subsequent blindness or severe visual impairment. The hereditary form of RB (40% of cases) is mostly bilateral and those patients are treated with enucleation and/or external beam radiotherapy (EBRT), and/or thermo-chemotherapy. Patients with unilateral RB are mostly treated with enucleation; after enucleation a prosthesis is inserted.

Considering the high survival rate of RB patients and the severe impact of the late effects of RB and its treatment, it is important to evaluate health-related quality of life (HRQoL) of RB survivors. So far, little attention has been paid to this subgroup of pediatric cancer survivors. Many studies have reported on QoL of pediatric cancer survivors but, to our knowledge, only Sheppard et al. (2005) have assessed the general QoL of young RB survivors [3]. Their study showed that mothers experience compromised QoL in their children (8–16 years), particularly with regard to their physical and psychosocial functioning, as compared to population norms. However, no study has yet made use of child self-reports, nor were any health-related questionnaires applied. Children are able to report reliably on their own well-being and functioning if the questionnaire is appropriate to the child's age and cognitive level[4]. Several factors that may jeopardize the HRQoL of RB survivors include, cosmetic deformities due to enucleation or EBRT[5,6], visual impairment[7,8], enhanced risk of second primary tumors [9,10] and, in heritable RB patients, the 50% potential for offspring with RB.

The aim of the present study is to investigate HRQoL of young Dutch RB survivors using a self-report and a proxy measure, and to evaluate potential effects of illness-related and demographic variables on HRQoL. Further, to compare HRQoL of the survivors with that of healthy matched individuals, controlling for age and gender. Finally, to compare the children's own evaluations of their HRQoL with that of their parents.

## Methods

### Study design

This population-based, cross-sectional study was performed between June 2005 and June 2006. It was approved by the ethics committees of the participating centers and was conducted according to the principles of the Helsinki declaration.

We consulted the Dutch national RB register [11] to collect personal data of all eligible RB survivors. The national Dutch RB register is unique because it is virtually complete from 1945 until 2006. Eligibility requirements for inclusion in this study were: (1) current age of the child between 8 and 18 years, (2) sufficient comprehension of the Dutch language in general, (3) treatment for RB at the VU University Medical Center (Amsterdam), at the University Medical Center of Utrecht, or at the University Medical Center St. Radboud (Nijmegen) (accounting for 98% of the national register), and (4) adequate intellectual level to understand the study questionnaires. On the basis of the national Dutch RB register, we excluded all survivors known to have mental retardation.

All survivors participated on the basis of written informed consent; for those aged less than 12 years parental agreement.

### Procedure

As soon as informed consent forms were received, parents were telephoned to make an appointment for a home visit. The KIDSCREEN questionnaires [12] were sent by post, with the instruction for the children to complete them alone and independently from other family members. In case of severe visual impairment of the children and/or parents, assistance was offered to fill in the questionnaires using an adapted computerized version. During the home visit we gathered socio-demographic information, discussed possible problems with completion of the KIDSCREEN questionnaire, and checked the completeness of it. We determined whether the children had completed the KIDSCREEN questionnaire themselves, by simply asking the children individually and their parents.

### Measures

#### Socio-demographic and illness-related factors

Data on the marital status of the parents were categorized as: single-parent family, or child living with both biological parents. RB-specific information collected from medical archives included heredity, type of treatment, and visual acuity. Conclusions on heredity of the disease were based on DNA research, bilateralism, and family history. Treatment strategies were categorized as: a) enucleation, b) EBRT, c) enucleation and EBRT, d) different combinations of chemotherapy and remaining therapies (thermo-chemotherapy, laser photocoagulation, plaque therapy and cryotherapy)[5]. Visual acuity was defined as visual acuity after subjective refraction in the survivor's best eye, and was categorized according to the WHO guidelines as: 1) normal vision (> 0.3), 2) low vision (0.05–0.3), and blindness (< 0.05) [13].

#### HRQoL measures

The KIDSCREEN-52 child/adolescent self-report instrument is a generic HRQoL questionnaire, developed within a European project [12,14]. The HRQoL questionnaire is designed to assess children's (8–11 years) and adolescents' (12–18 years) own perceptions of their subjective health and well-being.

The KIDSCREEN-52 proxy research instrument is derived from the above-mentioned self-report version, and designed to assess parental perceptions of their child's health and well-being [12].

The dimensions of HRQoL that are examined in the two versions of the KIDSCREEN are: "physical well-being", "psychological well-being: life satisfaction and positive emotions", "moods and emotions", "self-perception: body image and self-esteem", "autonomy", "parent relations and home life", "peer relations and social support", "cognitive and school functioning", "bullying and social acceptance", and "perceived financial opportunities". The recall period for most items is one week. The score of each dimension was calculated as the mean of the ratings of items that pertained to that dimension, after the score of each dimension was transformed linearly to a 0–100 point scale, with 100 indicating the best HRQoL and 0 the worst. Both instruments have shown acceptable reliability and validity coefficients [12]. Dutch population norms are available for both the child and parent version [12].

### Statistical analyses

Analyses were performed with SPSS 11.5 for Windows. Differences between (subgroups of) RB survivors and Dutch population norms were analyzed using one-sample t-tests. We also transformed these scores into z-scores. Possible predictors of the HRQoL subscales were studied by multiple regression analysis (backward elimination). Variables that were likely to affect the HRQoL subscales (dependent variables) were included in the regression model. These independent variables were: gender, age group (8–11 years or 12–18 years), marital status of parents, heredity, type of treatment, and visual acuity. Preliminary analyses showed interdependency of all illness-related factors (heredity, type of treatment, and visual acuity). Only visual acuity is included as an illness-related factor in the regression analysis, because this factor is a consequence of the disease with which the survivors were confronted daily. All tests were two-sided, with a 5% significance level. Paired group t-tests and Pearson's correlation coefficients were computed to measure how children's/adolescents' and parents' reports of HRQoL were related using the KIDSCREEN-52 self-report and the KIDSCREEN-52 proxy report, respectively. Correlation coefficients of >0.6, 0.4–0.6 and <0.4 were considered as strong, moderate and poor correlations, respectively.

## Results

### Participant's characteristics

From the national Dutch RB register, 99 RB survivors appeared to be eligible for our study; of these RB survivors, 4 (4%) could not be traced due to missing or incorrect personal data. Of the remaining 96, 67 RB survivors (70%) and their parents agreed to participate. After the home visit, we excluded 2 survivors for whom it was obvious (during personal communication) that they did not understand the questions well enough to fill in the questionnaire. Of the 28 traceable non-participating RB survivors, 18 (19%) refused to participate and 10 (11%) did not respond within the study period. Reasons for refusal of participation were mainly lack of time, lack of interest, or avoidance of confrontation with the disease. Patient characteristics between participants and non-participants did not differ significantly with regard to gender, age, heredity and type of treatment. No information was available on visual acuity, living situation, life events, and education of the non-participating children.

Table 1 presents socio-demographic and RB-related data on the 65 survivors. In addition to the data in Table 1, most hereditary RB survivors (83%) were bilaterally affected. Also, most hereditary RB survivors were treated with a combination of enucleation and EBRT (40%) whereas most non-hereditary RB survivors (91%) were treated with enucleation only. All unilaterally affected survivors had normal visual acuity in their non-affected eye. Of the bilaterally affected survivors, 8 (27%) had low vision and 2 (6%) survivors were blind. Of the survivors, 52 (80%) had an ocular prosthesis. In most cases (86%) the proxy informant was the mother. The mean age of the parents was 43.63 ± 6.5 years; 9 of them (6 fathers and 3 mothers, 14%) had suffered heritable RB themselves.

**Table 1:** Socio-demographic and RB-related information on the total group, and for RB survivors aged 8–11 years and 12–18 years separately

	RB survivor total group (N = 65)	RB survivors, children (N = 28)	RB survivors, adolescents (N = 37)
Age (yrs) *(mean (SD))*	12.7 (2.9)	9.8(0.9)	14.9 (1.6)
Age at diagnosis (yrs) *(mean (SD))*	1.6 (1.6)	1.2 (1.3)	1.8 (1.8)
Gender *(n (%))*			
*Female*	31 (48%)	15 (54%)	16 (43%)
Education *(n (%))*			
*Mainstream*	57 (88%)	23 (82%)	34 (92%)
*Special*	8 (12%)	5 (18%)	3 (8%)
Hereditary RB *(n(%))*			
*Non-hereditary RB*	35 (54%)	12 (43%)	23 (62%)
*Hereditary RB*	30 (46%)	16 (57%)	14 (38%)
Laterality RB *(n(%))*	40 (62%)	14 (50%)	26 (70%)
*Unilateral RB*			
Treatment RB *(n(%))*			
*Enucleation*	39 (60%)	14 (50%)	25 (68%)
*Radiotherapy*	10 (15%)	4 (14%)	6 (16%)
*Combi enucleation + radiotherapy*	13 (20%)	8 (29%)	5 (13%)
*Chemo/laser/plaque*	3 (5%)	2 (7%)	1 (3%)
Visual acuity *(n(%))*			
*Normal vision*	54 (83%)	22 (79%)	32 (86%)
*Low vision and blindness*	11 (17%)	6 (21%)	5 (14%)
Life events *(n(%))*			
*None*	18 (28%)	10 (36%)	8 (21%)
*Life events*	46 (71%)	18 (64%)	28 (76%)
*Missing*	1 (1%)		1 (3%)
Marital status parent *(n(%))*			
*Single-parent family*	20 (31%)	6 (21%)	14 (38%)
*Child living with both parents*	45 (69%)	22 (79%)	23 (62%)

### Group comparisons

#### A. Outcome measure: KIDSCREEN-52 child/adolescent self-report

##### A1. Comparison with Dutch reference groups

RB survivors reported significantly better HRQoL than the Dutch population-based reference group on the dimensions: "moods and emotions" (mean difference (MD) = 4.39: t[62] = 2.9, *p *= 0.005) and "autonomy" (MD = 2.27: t[62] = 2.1, *p *= 0.043).

**Figure 1:**
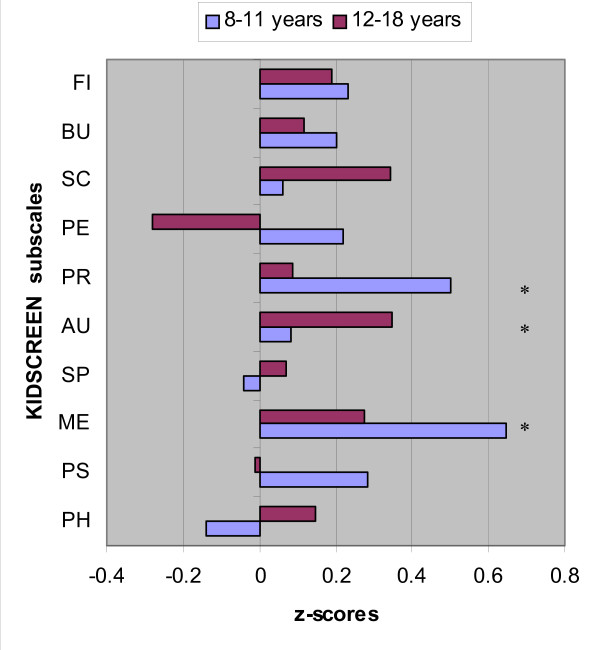
**Deviation from mean in the Dutch reference group (z = 0), expressed as a z score for RB children (8–11 yrs) and RB adolescents (12–18 yrs) separately**. KIDSCREEN-52 child/adolescent self-report subscales: FI = Financial; BU = Bullying; SC = School; PE = Peers; PR = Parent relations and home life; AU = Autonomy; SP = Self-perception; ME = Moods & Emotions; PS = Psychological well-being; PH = Physical well-being. Significance between Dutch reference group and RB children or adolescents: * p < 0.005.

Children (aged 8–11 years) surviving RB reported a better HRQoL on the dimensions "moods and emotions" (MD = 6.3: t[26] = 2.7, *p *= 0.011) and "parent relations and home life" (MD = 4.2: t[26] = 2.4, *p *= 0.023) than the age-matched reference group. Adolescent (aged 12–18 years) RB survivors reported a significantly better HRQoL on the dimension "autonomy" (MD = 3.1: t[35] = 2.1, *p *= 0.041). (see also Figure 1).

Irrespective of their age, female RB survivors reported better HRQoL than their female reference group on the dimensions "moods and emotions" (MD = 7.1: t[29] = 3.7, *p *= 0.001) and "school environment" (MD = 6.3: t[29] = 2.6, *p *= 0.015). No differences were found between boys surviving RB and their male reference group.

We further divided the group into male and female children, and male and female adolescents and compared those subgroups with their relevant reference group. Within the female group, young girls surviving RB reported better HRQoL than their relevant reference group on the dimension "moods and emotions" (MD = 7.6: t[13] = 2.7, *p *= 0.019), whereas adolescent female survivors reported better HRQoL on the dimension "moods and emotions" (MD = 5.8: t[15] = 2.3, *p *= 0.033), but also on the dimensions "autonomy" (MD = 4.4: t[15] = 2.1, *p *= 0.05), "school" (MD = 5.5: t[15] = 3.2, *p *= 0.005) and "financial" (MD = 4.9: t[15] = 2.3, *p *= 0.037) than their reference group. No significant differences were found in HRQoL between adolescent male RB survivors and their reference group.

##### A2. Within-group of RB survivors

**Figure 2:**
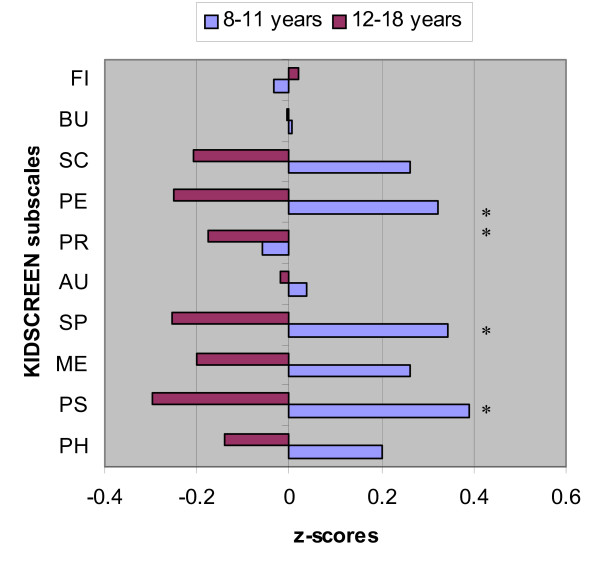
**Deviation from mean in the total RB group (z = 0), expressed as a z score for children (8–11 yrs) and adolescents (12–18 yrs) separately**. KIDSCREEN-52 child/adolescent self-report subscales: FI = Financial; BU = Bullying; SC = School; PE = Peers; PR = Parent relations and home life; AU = Autonomy; SP = Self-perception; ME = Moods & Emotions; PS = Psychological well-being; PH = Physical well-being. Significance between RB child survivors and adolescent RB survivors: * p < 0.005

From the multiple regression analyses, age was negatively associated with the dimensions "psychological well-being" (R^2 ^= 0.117, *p *= 0.006), "social support and peers" (R^2 ^= 0.083, *p *= 0.024) and "parent relations and home life" (R^2 ^= 0.110, *p *= 0.008). Adolescent RB survivors scored lower on all these subscales than the young survivors. Visual acuity (β = 0.243, *p *= 0.049) and age (β = 0.317, *p *= 0.011) were negatively associated with the HRQoL dimension "self-perception" (R^2 ^= 0.148, *p *= 0.009). RB survivors with normal visual acuity in their non-affected eye reported better HRQoL on the dimension "physical well-being" than visually impaired RB survivors (R^2 ^= 0.070, *p *= 0.038); see also Figure 2 and Table 2.

**Table 2:** Multiple regression analyses (backward elimination) for HRQoL dimensions by demographic and social factors, using the KIDSCREEN-52 self-report questionnaire and KIDSCREEN-52 proxy-report

Dependent variable	F	p	R^2^	Independent variable	β (standardized)
**KIDSCREEN-52 self-report**					

Psychological well-being	8.113	0.006	0.117	Age	-0.306
Self-perception	5.911	0.018	0.09	Age	-0.343
Parent relations and home life	7.569	0.008	0.110	Age	-0.299
Social support and peers	5.353	0.009	0.152	Life events	-0.332
					
**KIDSCREEN-52 proxy-report**					
Psychological well-being	3.908	0.05	0.06	Age	-0.245
Self-perception	3.920	0.05	0.063	Age	-0.252

#### B. Outcome measure: KIDSCREEN-52 proxy parent report

##### B1. Comparison with Dutch reference data

Parents' rating of the HRQoL of the RB surviving child did not differ significantly from ratings in the Dutch reference group[12]. Only parents of young boys surviving RB reported lower HRQoL scores on the dimension "autonomy" (MD = 3.5: t[11] = -2.7, *p *= 0.019).

##### B2. Within-group of parents of RB survivors

Age of the survivor was negatively associated with the dimensions "psychological well-being" (R^2 ^= 0.06, *p *= 0.05) and "self-perception" (R^2 ^= 0.063, *p *= 0.05). Parents of adolescent RB survivors reported lower HRQoL on these two subscales than the parents of young survivors. HRQoL ratings of parents of visually impaired RB survivors were better than those of parents of visually unimpaired survivors on the dimension "autonomy" (R^2 ^= 0.080, *p *= 0.026); see also Table 2.

#### C. Self-report vs. Parent Proxy report

Table 3 presents data on the correspondence between child and parent report. Survivors' perceptions and parents' perceptions correlated poorly on all dimensions (correlation coefficients ranged from 0.009 to 0.210, all *p*-values > 0.05). RB survivors perceived their HRQoL on the dimension "moods and emotions" to be better than their parents' assessment (MD = 5.9: t[59] = 2.6, *p *= 0.01).

**Table 3:** Correspondence between reports from the children and the parents: Pearson's correlation coefficients (PCC) and paired t-tests. Data are mean differences and (SD)

Kidscreen Domains	PCC (r)	Mean difference child vs. parent	*t*
Physical well-being	0.043	1.4 (0.1)	0.63
Psychologic well-being	0.142	1.4 (1.4)	0.78
Moods and emotions	0.141	5.8 (2.4)	2.62*
Self-perception	0.009	3.1 (1.0)	1.61
Autonomy	0.088	2.2 (1.2)	1.58
Parent relations and home life	-0.036	9.7 (48.4)	1.23
Social support and peers	-0.128	1.0 (1.5)	-0.51
School environment	0.176	1.7 (0.2)	0.84
Social acceptance	0.160	1.4 (1.4)	0.75
Financial resources	0.210	1.4 (0.7)	0.78

Significance: * p < 0.005.

## Discussion

The present study assessed the HRQoL of a unique Dutch population of young RB survivors using the KIDSCREEN questionnaire. To our knowledge, this is the first study to examine HRQoL in RB survivors with both a child and adolescent self-report, and a parent proxy-report questionnaire.

Our results suggest that the perceived HRQoL of children and adolescents who survived RB is not substantially different from the HRQoL of the normal population. It is noteworthy, however, that RB survivors report better "moods and emotions" and that they consider themselves to be more autonomous than healthy children. Parents of RB survivors report their child's HRQoL to be comparable with that of healthy children. This latter result is only partly in line with our earlier study, in which adult RB survivors were shown to experience a relatively good overall QoL, but a slightly worse mental health compared with a population-based reference group [15].

The discrepancy between adults and younger survivors might be caused by the fact that child and adolescent survivors are probably less well informed and/or are less aware of the effects in later life of the hereditary form of RB, such as risk for offspring, and enhanced risk for second primary tumors. During the home visits, some parents of hereditary RB survivors reported difficulties in informing their child about these effects, and were uncertain about the timing and the way the message should be conveyed. Shankar et al. (2005)also suggest that young (aged 8–12 years) childhood cancer survivors are too young to have encountered some of the negative psychosocial impacts of cancer and its treatment [16].

Studies assessing HRQoL of survivors of childhood cancer have, in general, reported contradictory results [17]. Our findings are in line with studies of Shankar et al. (2005), Pemberger et al. (2005), Apajasalo et al. (1996), Gray et al. (1992), Langeveld et al. (2004) and Maunsell et al. (2006), which report that long-term childhood cancer survivors show comparable or even higher-than-average positive subjective rating of the various areas of HRQoL [16-22]. According to these general childhood cancer studies, the observed excellent HRQoL could be explained in two ways. First, following the theory of response shift it may be that (as a result of the experience of cancer) the internal standards, values or conceptualizations of HRQoL change [23]. The experience of surviving cancer might lead to a better appreciation of being alive and to considering that possible impairments are of less importance. Barakat et al. (2006) found that a majority of adolescent childhood cancer survivors and their parents reported post-traumatic growth (PTG) [24]. Greater perceived treatment severity and life threat was associated with PTG. Diagnosis of cancer after age 5 years resulted in more perceived benefit and greater posttraumatic stress symptoms for adolescent survivors. Second, coping with the stress of the long-term effects of childhood cancer may have enhanced certain qualities of the survivors making them better able to cope with adversity in their lives.

It is conceivable that some of above-mentioned explanations may also apply to child and adolescent RB survivors. However, because this latter group often had treatment at a very young age, the time since treatment is long and the period of treatment in most cases was relatively short compared with most other forms of childhood cancer. This implies that, for this group, there may be less opportunity for the development of compensating abilities. In contrast to our results, other studies have reported a lower HRQoL for childhood cancer survivors in comparison with a healthy population [25-27]. Clearly, further research is necessary to assess the plausibility of the various explanations related to this group.

With respect to the survivor's own perceptions of their HRQoL, the following findings are of particular interest: especially adolescent RB survivors feel themselves to be more autonomous than healthy adolescent children, whereas younger RB survivors do not yet share this feeling. Younger RB survivors perceive themselves to be happier and as having closer relationships with their parents than children who did not experience RB. On closer inspection, increased ratings of HRQoL in RB survivors are mainly traceable to perceptions of adolescent girls who report themselves to be in a better mood, more autonomous, better at school and financially more independent than girls in the Dutch reference group. Thus the good HRQoL reported by RB survivors during childhood and early adolescence remains true, despite the fact that adolescents are generally more able to reflect on their own functioning and appearance. Indeed, in our group we found an overall significant negative effect of age regarding psycho-social well-being, possibly even concealing the remarkable increase of HRQoL in girls.

Our RB survivors with a normal vision reported better physical well-being than visually impaired RB survivors. This result is in line with a recent methodological study by Birch et al. (2007) which also confirmed the common expectation that children with more severe visual impairment experience poorer competence than those with only "unilateral" impairment [28]. In our study, parents of visually impaired RB survivors reported that their child had a higher level of autonomy than did parents of children with normal vision. Relatively good psychological adjustment in the more affected children may be caused by increased parental attention, as was observed in a sibling study [29].

With respect to parents' perceptions of their child's HRQoL, it is noteworthy that these are very different from the child's own perceptions. In general parents do not experience any difference in the HRQoL of their own child as compared to matched groups. Parents of young boys observed their son to be even less autonomous than their male peers. Within the RB group, parents of visually impaired RB survivors rated their child to be more autonomous than parents of RB survivors with normal vision. This finding may touch on a compensatory mechanism; however, we cannot exclude that visually impaired children undergo a different learning process whereby they learn to function better autonomously than children with normal vision. These findings are partly in concordance with Sheppard et al. (2005) who found that mothers reported lower levels of HRQoL for their children and adolescents treated for RB compared with population norms [5].

Some limitations of the present study should be addressed. First, the small number of survivors limits the statistical power of the study. Second, it is conceivable that some of the non-participating RB survivors experienced a different HRQoL. It is not clear, however, whether this would result in better or worse results. For example, patients who feel good might disregard the importance of the study and, on the other hand, parents of non-participating survivors may have refused participation to avoid their child being confronted with their disease again, which might suggest serious concern of the parents, or worse coping strategies and a poorer QoL. Third, the HRQoL of the survivors was only partly explained by the factors that were investigated in the present study. Other explanatory factors, such as coping and family functioning, may also be of importance; future studies should explore these factors. Indeed one would expect coping skills and HRQoL to interact, but unfortunately it was beyond the scope of this study to include such measures. Fourth, the results might be influenced by the choice of a general HRQoL questionnaire. In some specific cases our clinical impression seemed to be in contrast with the positive results of our study. An example we sometimes encountered in clinical practice is that RB survivors often have a different facial appearance due to the treatment; this may often lead to bullying and staring. In this study we found it important to compare our data with a healthy Dutch reference group, but a general HRQoL instrument (such as KIDSCREEN) only measures broad areas of HRQoL and may not identify such issues specifically associated with RB. In future studies it is advisable to use an RB-specific instrument or a vision-related HRQoL instrument besides a general HRQoL instrument. Unfortunately there is a shortage of such instruments, which in itself presents a challenge for future research.

## Conclusion

In conclusion, child and adolescent RB survivors report a good QoL compared with the Dutch reference group. It is noteworthy that perceptions of HRQoL as reported by parents and by children are very different. Parents judge the HRQoL of their child to be relatively poorer. Although the overall results are reassuring, other aspects of HRQoL that may have more specific relevance, such as psychological factors or coping skills, should also be explored in the future.

## Abbreviations

RB Retinoblastoma

HRQoL Health-Related Quality of Life

EBRT External Beam Radiation Therapy

WHO World Health Organization

MD Mean Differences

PTG Post Traumatic Growth

## Competing interests

The author(s) declare that they have no competing interests.

## Authors' contributions

JvD has coordinated the research, collected and analyzed the data and drafted the manuscript. SMI, ACM and JH participated in the design of the study, interpreted the data and revised the manuscript. AYNSM, PJR and PTCK interpreted the data and revised the manuscript. PDB contributed to the statistical analysis and revised the manuscript. All authors read and approved the final manuscript.
